# Developmental disorders among Norwegian-born children with immigrant parents

**DOI:** 10.1186/s13034-022-00547-x

**Published:** 2023-01-06

**Authors:** Hansen TM, Qureshi S, Gele A, Hauge LJ, Biele GP, Surén P, Kjøllesdal M

**Affiliations:** 1 Norwegian Institute of Public Health, Skøyen, Postbox 222, 0213 Oslo, Norway; 2 Institute of Public Health Science, Norwegian University of Lifesciences, Ås, Norway

**Keywords:** Developmental disorders, Immigration, Autism, ADHD

## Abstract

**Background:**

Risk of being diagnosed with different developmental disorders is found to vary with immigrant background. Knowledge about such differences in Norway are a starting point for equity in health services quality, and for early identification and prevention. Our objective was to assess the risk of receiving diagnoses of developmental disorders among children born in Norway (2006–2017) to two or one immigrant parent compared to children with two Norwegian-born parents.

**Methods:**

Information on developmental disorders was from the Norwegian Patient Register (NPR) and information on immigrant background, parental country of origin, parental education, and household income from Statistics Norway. We estimated hazard ratios (HR) with Cox proportional hazard regressions. With children with Norwegian background as reference category, we estimated HRs for immigration background and region of origin. All analyses were adjusted for sex, year of birth, parental education, and household income.

**Results:**

Children with two immigrant parents had a lower risk of receiving any developmental disorder diagnosis [HR 0.80 (95% CI 0.77, 0.82)] than children with Norwegian background, and lower risk of being diagnosed with attention deficit hyperactivity disorder (ADHD) diagnosis [HR 0.24 (95% CI 0.22, 0.27)], learning difficulties diagnosis [HR 0.39 (95% CI 0.33, 0.47)], and behavioral and emotional disorders [HR 0.52 (95% CI 0.49, 0.55)]. Children with immigrant parents had higher hazard than Norwegian background children of autism spectrum disorder (ASD) [HR 2.21 (95% CI 2.04, 2.39)], mental retardation [HR 1.84 (95% CI 1.64, 2.07)], language disorders [HR 1.30 (95% CI 1.20, 1.40)], and unspecified developmental disorders [HR 1.22 (95% CI 1.17, 1.28)]. Children with only one immigrant parent had lower risk of diagnoses than children of two immigrants.

**Conclusion:**

Risk of receiving a diagnosis of various developmental disorders varied substantially by immigrant background. Understanding the underlying mechanisms of these differences is warranted to ensure equity in health services and timely intervention.

**Supplementary Information:**

The online version contains supplementary material available at 10.1186/s13034-022-00547-x.

## Background

A considerable proportion of years lived with disability among younger children is due to impaired functioning in the physical, learning and language domains, which often come with developmental disorders [1]. Children with such disorders are at increased risk of suboptimal health and wellbeing. The specific causes of most developmental disorders are unknown. Known risk factors related to developmental disorders include genetics, both inherited and spontaneous mutations, environmental factors including birth complications and caesarian sections, obesity and diabetes during pregnancy, low intake of vitamins as well as exposure to heavy metals and other environmental toxins [2–4]. Common developmental disorders include behavioral and emotional disorders in childhood, including attention deficit hyperactivity disorder (ADHD), autism spectrum disorders (ASD), learning difficulties and language disorders.

An estimated 0.7% of children in Norway are diagnosed with ASD [5], and 3–5% of children have an ADHD diagnosis [6]. The risk of receiving an ASD diagnosis has in several studies been shown to be higher among children of immigrants than among other children, especially for low-functioning ASD [7–11]. Evidence is inconclusive about the relationship between receiving an ADHD diagnosis and immigrant background [10], but in Sweden receiving an ADHD diagnosis is associated with social disadvantage [12], and in Finland with having immigrant parents [13]. Children with one or two immigrant parents have also been reported to have a higher risk of developmental disorders related to speech and language, academic skills, or coordination [14, 15]. Differences by immigration background in diagnoses of developmental disorders could reflect both differences in prevalence, due to exposure to various environmental risk factors or genetics, or due to differences in use of health services and in being diagnosed timely and correctly [10].

In Norway, 12% of children under the age of 18 are born to two immigrant parents [16], and an increasing number of children live in exogamous families; i.e., with one immigrant and one native-born parent. Knowledge about differences in risk of diagnoses of developmental disorders among children by immigration background in Norway is warranted for early identification, prevention, and equity in health care service quality.

This article explores and describes how diagnoses of developmental disorders vary between children with Norwegian and with immigrant backgrounds. To do this, we used linked Norwegian register data to assess the risk of receiving diagnoses of developmental disorders among children born in Norway between 2006 and 2017 to two or one immigrant parent compared to children with two Norwegian-born parents.

## Methods

### Study population

The study population included all children born in Norway between 2006 and 2017 and registered in the Medical Birth Registry of Norway (MBRN). Number and year of diagnoses for developmental disorders registered in the Norwegian Patient Register (NPR) and information on immigrant background, parental country of origin, parental education and household income from Statistics Norway were linked by the child’s national personal identification number.

Children with missing information on immigration category or regional background (N = 77 243), those registered as emigrated or unknown status (N = 82 987), stillborn or dead before the age of 2 (N = 50 839), or with missing information on parental education and/or household income (N = 817) were excluded. Children whose registered immigrant status did not correspond to parents` country of birth, e.g., children registered as born in Norway to two Norwegian born parents, but where one or two parents had another birth country than Norway, were excluded (N = 37 875). After all exclusions, the total sample included 669 770 children.

### Variables

Children born to two native-born parents are referred to as having “Norwegian background”, whilst those born to at least one foreign-born parent as having an “Immigrant background”. Children with two foreign-born parents are referred to as having “immigrant parents”. Those with a foreign-born mother and native-born father are referred to as having “immigrant mother”, and correspondingly, we use “immigrant father” when referring to those with a foreign-born father and native-born mother.

Parental regional background was defined by parental country of birth. Among children with immigrant background, region of origin was classified according to national standards [17] into the following categories: “EU/European Economic Area (EEA), Oceania, United States of America (USA), and Canada”, “Europe except EU/EEA”, “Asia”, “Africa”, and “Latin America”. Relatively few children were registered with a parental regional background from USA/Canada or Oceania. These regions were consequently grouped into one category with the EU/European Economic Area, considered most similar with respect to socio-economic development. Grouping based on socio-economic development is useful because of known associations between socioeconomic status and neurodevelopmental disorders. The “Europe except EU” category includes mostly eastern European countries such as Kosovo, Russia, Bosnia and Herzegovina, Serbia, and North Macedonia. Most children with two immigrant parents had parents with the same regional background (93%). Where there was a discrepancy between maternal and paternal regional background, region of origin is defined as the mother`s region of origin.

Parental education was recorded as highest attained educational level throughout the study period by either parent and categorized into “primary school” (≥ 9 years), “upper secondary school” (10–12 years), “university/university college, lower” (13–16 years) and “university/university college, higher” (≥ 17 years). Household income was recorded as yearly household income (in NOK) after tax, divided by number of consumptions units (EU-scale) in the household. Household income groups were calculated based on the tertials of household income per year and included in the analyses as household income group at year of birth.

The Norwegian Patient Registry contains ICD-10 diagnoses given in secondary and tertiary health care (both inpatient and outpatient) from 2008 onwards. We included 8 diagnostic categories of developmental disorders (ICD-10 codes in Additional file 4: Table S1): “mental retardation”, “language disorders”, “learning difficulties”, “other developmental disorders”, “ASD”, “ADHD”, “behavioral and emotional disorders in childhood”, and “unspecified developmental delay”. We use the term “any developmental disorder” as a summative category that describes children who received at least one of these eight developmental disorders. Children with a diagnosis within a diagnostic category at least once during the specified time frame were classified as having the respective diagnosis.

### Analyses

The number and percentage receiving each diagnosis according to parent’s immigration category and region of origin was reported. In Cox proportional hazard regressions, hazard ratios (HR) with 95% confidence intervals for each diagnosis were calculated for immigrant category (having an immigrant mother, father, or both) and regional background, compared to having parents with Norwegian background. We compared regional background for all children with an immigrant background (either mother, father, or both) to children with a Norwegian background. Analyses were adjusted for sex and year of birth, parental education, and household income.

Each participant was followed from 2008 or year of birth (if later than 2008) until first diagnosis per diagnostic category, the end of 2018 or year of death. Children were between 0 and 12 years during follow up. We made additional, separate analyses for diagnoses given before the age of 6 years, which in Norway is the age children start school, and reported mean age per diagnostic category by regional background.

Sensitivity analyses for association of diagnosis with regional background were carried out for children with two immigrant parents, with an immigrant mother and an immigrant father separately.

Analyses were performed in R [18], using the ‘survival’ package [19] for the analyses, and ‘forestmodel’ package [20] to create the figures.

## Results

Of the included children, 37% had at least one immigrant parent, of whom 51% were born to two immigrant parents, 27% to an immigrant mother and 22% to an immigrant father. Children with an immigrant background more often had parents in the lowest educational category compared to children with a Norwegian background. Most prominently among children with two immigrant parents, of whom 25% had parents in the lowest educational category (Table 1). The proportion with parents in the lowest educational group varied by region of origin and was highest among those with background from Africa and Asia. For all children with an immigrant background, children with parental regional background from EU/EEA/Oceania/USA/Canada had a higher proportion within the highest educational category, also compared to those with a Norwegian background (Table 1). The proportion with low household income was highest among children with two immigrant parents, and among these, highest among those with parental regional background from Africa or Asia. The highest proportion within the highest household income category was for those with one immigrant parent with background from EU/EEA/Oceania/USA/Canada.

**Table 1:** Characteristics of the sample, Norwegian-born children aged 0–18 years between 2006 and 2018, by parental immigration category and parental region of origin

	Two Norwegian-born parents	Two immigrant parents	EU/EEA/Oceania/USA/Canada	Europe except EU	Africa	Asia	Latin America
Total, N	487 545	93 432	27 802	10 438	19 743	34 197	1 252
Parental education, %							
Primary	4.9	25.2	8.7	17.2	42.8	29.8	14.4
Secondary	29.9	27.3	25.8	33.5	26.6	27	25.4
Higher, low	42.8	25.9	30	26.3	20.3	26.1	25
HIgher, high	22.5	21.5	35.5	22.9	10.3	17.1	35.3
Household income (tertiles), %							
Low	28.9	73	62.9	65	89.9	74.7	58.8
Middle	38.7	18.5	24.5	24.3	8.3	17.5	23
High	32.4	8.5	12.6	10.7	1.9	7.8	18.2

	Only immigrant mother	EU/EEA/Oceania/USA/Canada	Europe except EU	Africa	Asia	Latin America
Total, N	49,503	22,268	4463	2312	15,921	4539
Parental education, %						
Primary	7.7	4.5	5.4	10	12.7	6.9
Secondary	27.9	22.4	20.6	32.7	35.6	33
Higher, low	33.9	35.4	30.5	34.3	33.1	32.4
HIgher, high	30.4	37.6	43.6	23.0	18.6	27.7
Household income (tertiles), %						
Low	39	29.0	35.1	51.2	51.3	43.1
Middle	31.5	33.3	33.8	27.5	28.9	30.9
High	29.5	37.7	31.1	21.4	19.8	26

	Only immigrant father	EU/EEA/Oceania/USA/Canada	Europe except EU	Africa	Asia	Latin America
Total, N	39 290	23 887	1 696	3 493	7 481	2 733
Parental education, %						
Primary	8.3	4.9	14.7	11.3	16.5	7.3
Secondary	22.2	18.9	34.5	21.5	31.0	21
Higher, low	38.9	40.1	34.5	41.9	34.1	40.2
HIgher, high	30.6	36.1	16.4	25.2	18.4	31.5
Household income (tertiles), %						
Low	40.7	31.2	47.6	61.5	55.8	52
Middle	32.4	34.5	36.3	26.4	28.1	31.3
High	26.9	34.4	16.1	12	16.1	16.7

The proportion of children with any developmental disorder was lower among children with an immigrant background compared to children with a Norwegian background (Table 2). The most common diagnoses for children with a Norwegian background were ADHD, behavioral and emotional disorders, and unspecified developmental delay.

**Table 2:** Developmental disorder diagnoses given in secondary/tertiary health care [N (%)] and between 2008 and 2018 among children born in Norway between 2006 and 2017 with two Norwegian-born parents, two immigrant parents and one immigrant parent (mother/father)

	Two Norwegian-born parents	Two immigrant parents	EU/EEA/Oceania/USA/Canada	Europe except EU	Africa	Asia	Latin America
Mental retardation	1332 (0.27)	495 (0.53)***	55 (0.20)*	51 (0.49)***	161 (0.82)***	224 (0.66)***	4 (0.32)
Language disorders	3594 (0.74)	974 (1.04)***	199 (0.72)	123 (1.18)***	216 (1.09)***	412 (1.20)***	24 (1.92)***
Learning difficulties	1762 (0.36)	144 (0.15)***	20 (0.07)***	18 (0.17)**	32 (0.16)***	67 (0.20)***	7 (0.56)
Other developmental disorders	2741 (0.56)	464 (0.50)*	81 (0.29)***	64 (0.61)	127 (0.64)	185 (0.54)	7 (0.56)
Autism spectrum disorders	2624 (0.54)	1111 (1.19)***	202 (0.73)***	125 (1.20)***	309 (1.57)***	462 (1.35)***	13 (1.04) *
ADHD	8873 (1.82)	461 (0.49)***	115 (0.41)***	52 (0.50)***	126 (0.64)***	150 (0.44)***	18 (1.44)
Behavioral and emotional disorders in childhood,	16338 (3.35)	1558 (1.67)***	342 (1.23)***	165 (1.58)***	312 (1.58)***	710 (2.08)***	29 (2.32)
Unspecified developmental delay	11218 (2.30)	2916 (3.12)***	565 (2.03)**	310 (2.97)***	686 (3.47) ***	1322 (3.87)***	33 (2.64)
Any developmental disorder	35794 (7.34)	5710 (6.11)***	1161 (4.18)***	633 (6.06)***	1340 (6.79)**	2485 (7.27)	91 (7.27)

	Norwegian-born father and immigrant mother	EU/EEA/Oceania/USA/Canada	Europe except EU	Africa	Asia	Latin America
Mental retardation	131 (0.26)	43 (0.19)*	13 (0.29)		55 (0.35)	16 (0.35)
Language disorders	439 (0.89)***	165 (0.74)	54 (1.21)***	18 (0.78)	142 (0.89)*	60 (1.32)***
Learning difficulties	115 (0.23)***	54 (0.24)**	12 (0.27)		37 (0.23)**	8 (0.18)
Other developmental disorders	260 (0.53)	119 (0.53)	28 (0.63)	13 (0.56)	80 (0.50)	20 (0.44)
Autism spectrum disorders	420 (0.85)***	161 (0.72)***	53 (1.19)***	20 (0.87)*	143 (0.90)***	43 (0.95)***
ADHD	548 (1.11)***	290 (1.30)***	62 (1.39)*	24 (1.04)**	104 (0.65)***	68 (1.50)
Behavioral and emotional disorders in childhood,	1405 (2.84)***	655 (2.94)***	145 (3.25)	76 (3.29)	362 (2.27)***	167 (3.68)
Unspecified developmental delay	1231 (2.49)**	508 (2.28)	116 (2.60)	44 (1.90)	447 (2.81)***	116 (2.56)
Any developmental disorder	3363 (6.79)***	1485 (6.67)***	348 (7.80)	152 (6.57)	1009 (6.34)***	369 (8.13)*

	Norwegian-born mother and immigrant father	EU/EEA/Oceania/USA/Canada	Europe except EU	Africa	Asia	Latin America
Mental retardation	133 (0.34)*	61 (0.26)		21 (0.60)***	36 (0.48) **	11 (0.40)
Language disorders	297 (0.76)	170 (0.71)	14 (0.83)	24 (0.69)	69 (0.92)	20 (0.73)
Learning difficulties	111 (0.28)*	61 (0.26)**	7 (0.41)	14 (0.40)	20 (0.27)	9 (0.33)
Other developmental disorders	188 (0.48)*	94 (0.39)***	8 (0.47)	16 (0.46)	54 (0.72)	16 (0.59)
Autism spectrum disorders	268 (0.68)***	152 (0.64)*	13 (0.77)	28 (0.80)*	56 (0.75)*	19 (0.70)
ADHD	561 (1.43)***	333 (1.39)***	24 (1.42)	76 (2.18)	77 (1.03)***	51 (1.87)
Behavioral and emotional disorders in childhood,	1192 (3.03)***	703 (2.94)***	52 (3.07)	122 (3.49)	226 (3.02)	89 (3.26)
Unspecified developmental delay	920 (2.34)	482 (2.02)**	44 (2.60)	73 (2.09)	248 (3.31)***	73 (2.67)
Any developmental disorder	2638 (6.71)***	1496 (6.26)***	115 (6.78)	253 (7.24)	568 (7.59)	206 (7.54)

N < 5 not shown. Star indicting difference to having two Norwegian-born parents: ***p < 0.001 **p < 0.01 *p < 0.05

The proportions with diagnoses of learning difficulties, ADHD, behavioral and emotional disorders, and other developmental disorders were lower among children with immigrant background compared to children with a Norwegian background, but with some variations by regional background (Table 2). For ASD and language disorders, a higher proportion of children with immigrant parents were diagnosed compared to children with a Norwegian background (Table 2). The higher proportions with an ASD diagnosis were consistent over regional backgrounds.

**Fig. 1:**
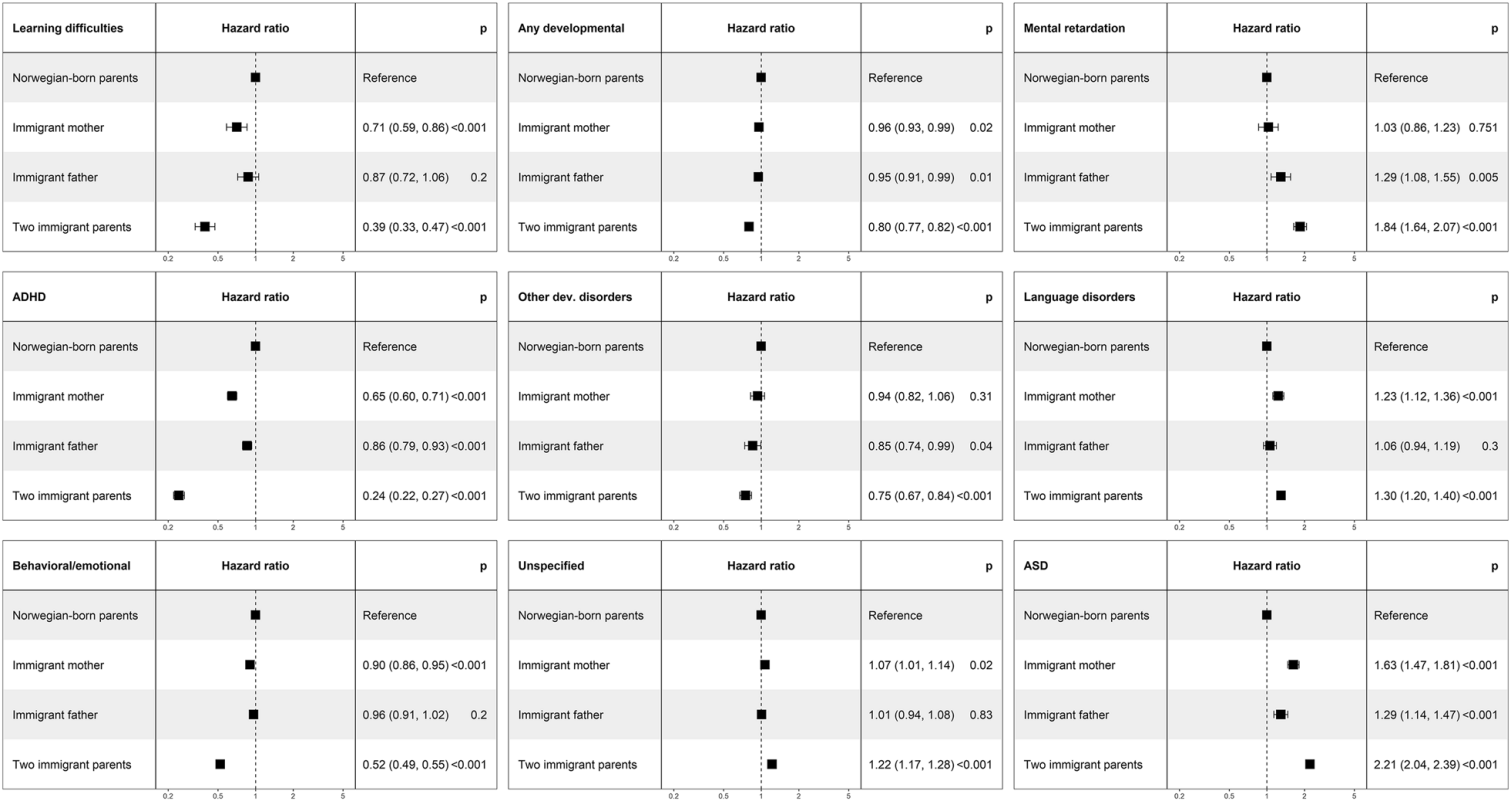
Hazard ratio for diagnosis of developmental disorder by parental immigration category for children born in Norway between 2006 and 2017, adjusted for birth year, sex, highest achieved parental educational level, and household income group, (95% confidence interval)

**Fig. 2:**
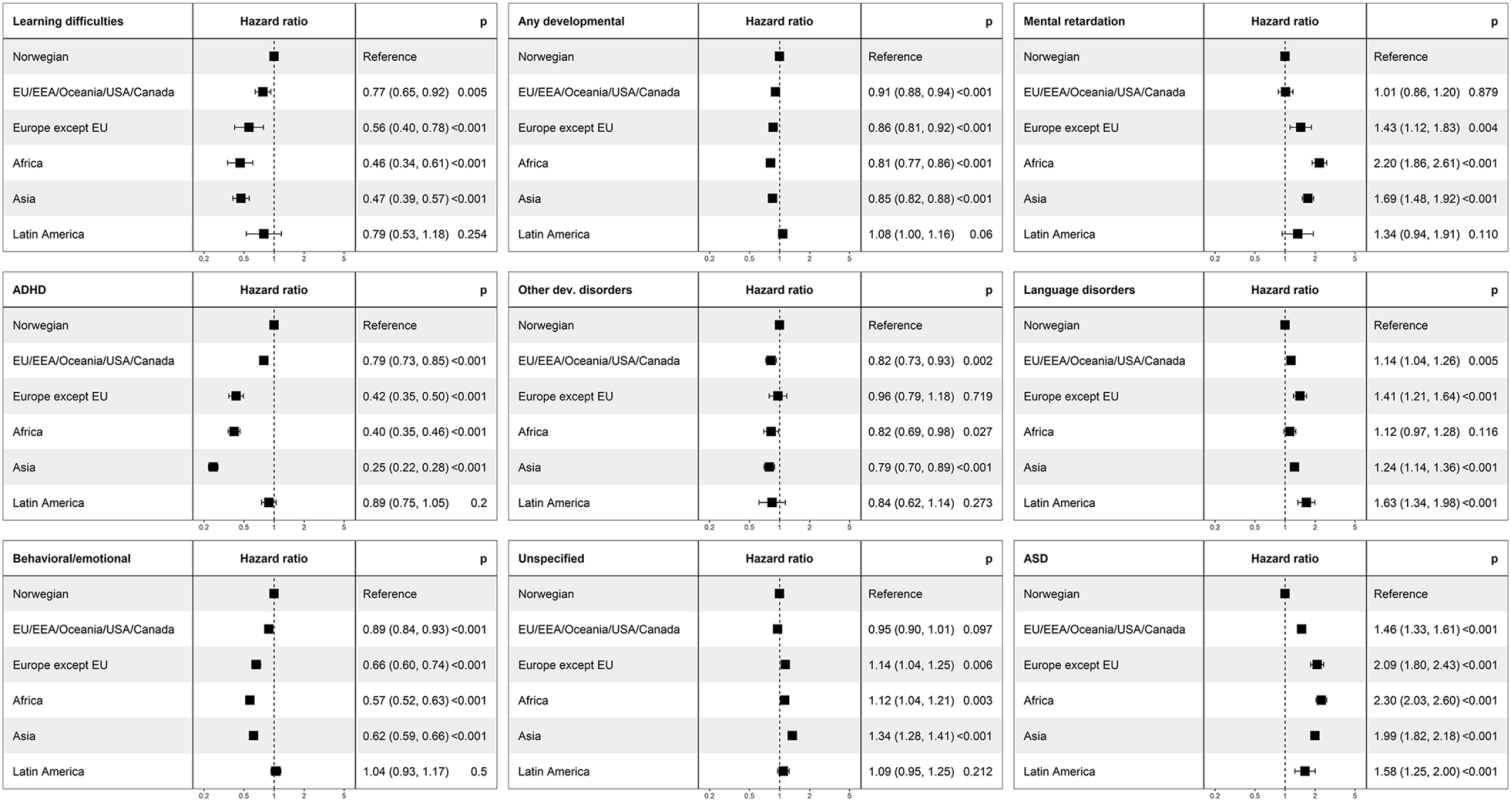
Hazard ratio for diagnosis of developmental disorder by parental regional background for children born in Norway between 2006 and 2017. Regional background for children with an immigrant background (either mother, father, or both) compared to children with a Norwegian background, adjusted for birth year, sex, highest achieved parental educational level, and household income group (95% confidence interval)

Results from cox regressions adjusted for sex, birth year, highest parental education and household income showed lower hazards of diagnosis of ‘any developmental disorder’ among children with an immigrant background [immigrant parents: HR 0.80 (95% CI 0.77, 0.82), immigrant mother: HR 0.96 (0.93, 0.99), immigrant father: HR 0.95 (0.91, 0.99)] compared to those with a Norwegian background. Differences between children with an immigrant and a Norwegian background were most prominent for diagnosis of ADHD [immigrant parents: HR 0.24 (95% CI 0.22, 0.27), immigrant mother: HR 0.65 (0.60, 0.71), immigrant father: HR 0.86 (0.79, 0.93)], learning difficulties [immigrant parents: HR 0.39 (0.33, 0.47), immigrant mother: HR 0.71 (0.59, 0.86)], and behavioral and emotional disorders [immigrant parents: HR 0.52 (95% CI 0.49, 0.55), immigrant mother: HR 0.90 (0.86, 0.95)] (Fig. 1). By region, the hazard for diagnoses of these disorders were generally lower among children with any other regional background when comparing to children with a Norwegian background, with some exceptions. Children with parental regional background from Latin America did not have a significantly lower hazard ratio for diagnoses of any of these disorders. Compared to children with a Norwegian background, children with parental regional background from Asia, Africa and Europe except EU stand out with the lowest hazard ratios for diagnoses of learning difficulties, ADHD, or behavioral/emotional disorder (Fig. 2).

Adjusted cox regressions showed higher hazards of diagnoses of ASD [immigrant parents: HR 2.21 (95% CI 2.04, 2.39), immigrant mother: HR 1.63 (1.47, 1.81), immigrant father: HR 1.29 (1.14, 1.47)], mental retardation [immigrant parents: HR 1.84 (1.64, 2.07), immigrant father: HR 1.29 (1.08, 1.55)], language disorders [immigrant parents: HR 1.30 (1.20, 1.40), immigrant mother: HR 1.23 (1.12, 1.36)], and unspecified developmental disorders [immigrant parents: HR 1.22 (1.17, 1.28)] among children with an immigrant background than among children with a Norwegian background (Fig. 1). For children with immigrant background, hazards were higher for a mental retardation or unspecified developmental disorder diagnosis among those with a parent from Europe except EU, Africa or Asia, for a language disorders diagnosis among those with a parent from Europe except EU or Latin America, and for an ASD diagnosis among those with parents from any of the regions compared to children with a Norwegian background (Fig. 2).

When assessing only diagnoses given before the age of 6, main findings of higher proportions of ASD and language disorders diagnoses, and lower proportions of ADHD diagnoses, among children with an immigrant background compared to children with a Norwegian background are consistent (Additional file 4: Table S1). In contrast to proportions with a diagnosis in the total sample, i.e., before the age of 12, a larger proportion of children with an immigrant background had a diagnosis for ‘any developmental disorder’ before the age of 6 compared to children with a Norwegian background.

Mean age for any developmental disorder diagnosis is considerably lower for children with two immigrant parents than for children with parents born in Norway (mean age 3.9 (SD 2.7) for children with two immigrant parents, 5.5 (SD 3.2) for children with Norwegian-born parents). Mean age for first diagnosis of ASD and mental retardation was lower among children with immigrant background, and for behavioral and emotional disorders higher, compared to children of a Norwegian background (Additional file 4: Table S3).

Figures presenting hazard ratios by regional background among children with an immigrant mother, immigrant father or immigrant parents separately compared to Norwegian-born parents are shown in Additional file 1: Figure S1, Additional file 2: Figure S2, Additional file 3: Figure S3. For ASD diagnosis, the association with parental regional background was stronger for having an immigrant mother than an immigrant father (Additional file 1: Figure S1 and Additional file 2: Figure S2). For children with only one immigrant parent, maternal regional background from Europe except EU and Latin America was associated with higher hazard of language disorders diagnoses and for Asia and EU/EEA/Oceania/USA/Canada also ASD diagnoses. Maternal regional background from Asia was associated with lower hazards of diagnoses of learning difficulties, ADHD, and behavioral and emotional disorders in childhood. These associations were not as prominent for those with an immigrant father only. For children with only one immigrant parent, hazard of mental retardation diagnosis was higher among those with paternal regional background from Africa, Asia and Latin America, an association which was not found for maternal regional background.

## Discussion

### Main findings

When assessing the risk of developmental disorders within any of the selected diagnostic categories, children with an immigrant background had a lower risk of diagnosis than children with a Norwegian background. For two of the overall most common diagnosis groups, behavioral and emotional disorder and ADHD, the risk of a diagnosis was lower among children with an immigrant background than among children with Norwegian background. However, children with an immigrant background had higher risk of receiving an ASD diagnosis than children with non-immigrant parents. Children with two immigrant parents also had higher risk than children with a Norwegian background of a diagnosis of mental retardation, language disorders or unspecified developmental delay. In addition to higher risk of an ASD diagnosis, children with an immigrant father had higher risk of a mental retardation diagnosis, and those with an immigrant mother of language disorders, compared to Norwegian background children. The differences in ASD prevalence between children with and without immigrant background was most pronounced for children under the age of 6 years.

A lower risk of diagnosis of any developmental disorder among children with immigrant parents than among those with Norwegian background could indicate lower true prevalence. However, it could also reflect variation in interpretation of symptoms from parents, teachers and other care givers, or variation in healthcare seeking behaviour, where some groups may be less likely than others to seek health care for such disorders. The latter hypothesis is consistent with our finding that immigrant groups have higher prevalence of more severe disorders that cannot be ignored, such as autism, and lower prevalence with less severe disorders that are harder to diagnose, such as ADHD.

### ADHD

Children with an immigrant background had lower odds of being diagnosed with ADHD than children with Norwegian-born parents, in contrast to studies from Finland, where children of immigrants were more likely than others to be diagnosed with ADHD [13]. In Sweden, children with parents from low- and middle-income countries have been found to have lower levels of ADHD medication than children with two Swedish born parents, whereas children of parents from high-income countries, or with one immigrant and one Swedish-born parent, had the same level of ADHD medication as children with two Swedish-born parents [21]. Environmental factors most consistently associated with ADHD are pre- and perinatal factors, such as severe early deprivation and psychological stress during pregnancy, low birth weight, and prematurity. It is, however, not clear that children with immigrant parents should experience lower levels of risk factors for ADHD. In general, one might expect immigrant mothers to experience greater psychological stress than Norwegian-born mothers. Further, mothers from Africa and parts of Asia often give birth to babies with lower birth weight than Norwegian-born mothers [22]. Within homogeneous populations from industrialized countries ADHD has a genetic component, but its importance for the differences between children with and without immigrant parents are unknown. Finally, differences in diagnosis may be related to use of health services, differences in tolerance or interpretation of symptoms and in how diagnoses are given.

### Autism spectrum disorders

The odds of receiving an ASD diagnosis were higher among children with at least one immigrant parent compared to children with two Norwegian born parents. It was highest among those with two immigrant parents, and higher among those with one immigrant mother than among those with one immigrant father. This is in line with findings from Finland, where children with two immigrant parents or an immigrant mother, but not those with an immigrant father, had higher risk of an ASD diagnosis than children with two Finnish born parent [9]. In Sweden, having a mother born outside Sweden, irrespective of father`s immigrant status, has been associated with risk of an ASD diagnosis [23]. Also, in Denmark, having a mother born outside Europe, and/or parents born in different countries, has been associated with increased risk of an ASD diagnosis [24]. Previous research has suggested that risk of ASD [25], and low functioning ASD especially [26], is associated with migration background from low HDI (human development index) countries, but not from high HDI countries. Our results do not reflect these observations, as risk of an ASD diagnosis was increased across all regions for those with two immigrant parents, for those with mothers from all regions except Africa, and those with fathers from EU/EEA/Oceania/USA/Canada, Europe except EU, and Africa. Several studies report that children with immigrant parents have higher risk of low-functioning ASD, but lower risk of high-functioning ASD [10]. We did not have data to assess such differences in our study, but as early diagnosis may be an indication of stronger symptoms, more pronounced differences for children of immigrants for early diagnosis could possibly reflect this.

Differences between children with and without immigrant parents in risk of receiving an ASD diagnosis can be related to both higher prevalence of ASD among children of immigrants than other children, and to differences in the likelihood of being correctly diagnosed with ASD. ASD is caused by genetic and environmental factors or a combination of the two [3]. Because the heritability estimates for ASD are based on homogeneous populations and explain variations between individuals, they carry little information about potential genetic causes for the observed prevalence differences between children with and without immigrant background. Rates of ASD are high among children with East African background in Norway [27]. These numbers do not correspond to reported rates of ASD among children in Eastern Africa [28], which could be due to several environmental factors including underdiagnosis in countries of origin, different environmental factors in Norway and stressors associated with growing up in an immigrant context. Indeed, a prior literature review highlights the importance of maternal migration stressors for the risk of ASD among their children [29]. Consistently, a study in Sweden found that the risk of ASD was higher if the mother had immigrated within 5 years of the birth compared to more than 15 years [26]. Overall, current research has identified several broad factors associated with ASD diagnosis in children with immigrant background, yet there remains a need for well-designed research about environmental causes for ASD among immigrants in Norway.

Differences in risk of diagnoses of developmental disorders may also be due to differences in health care seeking behavior of parents and bias in referral and diagnosis within health care services. There is evidence that health personnel are more likely to ignore symptoms of ASD or attribute them to other social or language problems among children with immigrant background [10]. Parents from some immigrant groups may also have less knowledge about symptoms of for instance ADHD or ASD or having children with such disorders may be considered shameful. Moreover, immigrant parents may face barriers to seeking health care, including low health literacy and knowledge about the health care system, low proficiency in the host language and practical barriers. All these factors could lead to underdiagnoses of developmental disorders among children of immigrants, and we thus hypothesize that our results reflect a true higher prevalence of ASD among immigrants, rather than a higher proportion with ASD being diagnosed, and that differences in prevalence possibly are larger than our results indicate.

### Language disorders

In our study, children with two immigrant parents or an immigrant mother had higher odds for a diagnosis of language disorders than children with two Norwegian parents. In Finland, having an immigrant mother, an immigrant father, or two immigrant parents have been associated with a diagnosis of speech and language disorder, most strongly among those with two immigrant parents, but stronger among those with an immigrant mother than an immigrant father [15]. Stronger associations with having an immigrant mother than father both in Finland and in our study, suggest that mothers are more important for language learning and development among children than fathers. Given the children with immigrant background typically grow up bilingual, bilingual language development is somewhat delayed and more variable, and diagnosis of language disorders in bilingual children is complicated, a higher prevalence of language disorders among children with immigrant background is to be expected [30, 31].

### Strengths and limitations

We used Norwegian registers with national and complete data on diagnoses given in secondary and tertiary health care for all children born in Norway between 2006 and 2017. The personal identification number per individual made it possible to link diagnoses from the Norwegian Patient register with data on parental education and household income from Statistics Norway. Our data, however, pertains only to diagnoses given in secondary and tertiary health care and does not include diagnoses given in primary care. For most developmental disorders among children, diagnoses are first set in secondary/tertiary care. Access to and use of secondary/tertiary care may be impacting diagnosis rates.

Children were followed from birth, or for those born in 2006 or 2007 from the age of 1 or 2 years. Very few developmental diagnoses are set before the age of 2, so left censoring should not be a problem. We had data until 2018, and children were followed up to a maximum of 12 years. Some children may receive a developmental disorder diagnosis at an age older than 12 years. Thus, differences in hazards could differ if we had been able to include a wider age group, and the mean age at diagnosis possibly higher. This is most relevant for the diagnoses ADHD and learning difficulties. Moreover, not all children were followed to the age of 12. We attempted to account for this by adjusting for year of birth in the regression analyses.

Further, as the Norwegian patient register started to register diagnoses in 2008, diagnoses registered this year might not be the first time of a diagnosis for a child, and thus the estimated mean age of diagnosis captured by the data may be too high. We might also have missed diagnoses given before 2008 if the child did not have any follow up care in secondary/tertiary care the following years. Further, we did not stratify immigrants by country of origin, which may downplay the difference in prevalence of different disorders between different groups.

Our data included only diagnostic categories and number of diagnoses given per category per year. We were therefore not able to differentiate between rates of specific codes within each category, for example differentiating between severity of the disorder. The analyses also assessed the rates and hazards of each diagnostic category separately. It is not uncommon for children to be diagnosed with multiple developmental disorders, for example a combination of both ASD and ADHD [32]. While our analyses did not take this into account, the data show that while there is a high rate of ADHD diagnoses among children with ASD, the opposite is not the case. For very young children, symptoms of developmental disorders may overlap and thus diagnoses may change over time [32].

### Implications

Studies seeking to understand risk factors leading to higher prevalence of ASD and language disorders among immigrants will help contribute to early intervention and diagnosis of these disorders. Especially for ASD, early interventions are crucial. It will also be important to understand the lower risk of diagnoses of ADHD, learning difficulties, and behavioral and emotional disorders in childhood among children with an immigrant background than among others, whether it is related to underdiagnosis or to protective factors. Practitioners should be aware of the reported differences in their work with children with an immigrant background.

## Conclusions

Risk of receiving a diagnosis of various developmental disorders varied by immigrant background. Understanding the underlying mechanisms of these differences is warranted to ensure equity in health services and timely intervention.

## Supplementary Information

**Additional file 1: Figure S1.** Hazard ratio for diagnosis of developmental disorder by maternal regional background for children born in Norway between 2006 and 2017 with only one immigrant parent (mother) compared to children with two Norwegian-born parents, adjusted for birth year, sex, highest achieved parental educational level, and household income group (95% confidence interval).

**Additional file 2: Figure S2.** Hazard ratio for diagnosis of developmental disorder by paternal regional background for children born in Norway between 2006 and 2017 with only one immigrant parent (father) compared to children with two Norwegian-born parents, adjusted for birth year, sex, highest achieved parental educational level, and household income group (95% confidence interval).

**Additional file 3: Figure S3.** Hazard ratio for diagnosis of developmental disorder by parental regional background for children born in Norway between 2006 and 2017 with two immigrant parents compared to children with two Norwegian-born parents, adjusted for birth year, sex, highest achieved parental educational level, and household income group (95% confidence interval).

**Additional file 4: Table S1.** ICD-10 codes included per diagnostic category. **Table S2.** Developmental disorder diagnoses given before the age of 6 in secondary/tertiary health care (N (%)) and between 2008 and 2018 among children born in Norway between 2006 and 2017 with two Norwegian-born parents, two immigrant parents and one immigrant parent (mother/father). **Table S3.** Mean age (year, SD) at diagnosis of developmental disorders given between 2008 and 2018 among children born in Norway between 2006 and 2017.

## Data Availability

The datasets generated and analyzed during this study are not publicly available due to data regulations but can be obtained from registry owners.

## Abbreviations

ADHD: Attention deficit hyperactivity disorder
ASD: Autism spectrum disorder (ASD)
EEA: European economic area
EU: European Union
HR: Hazard ratio
ICD-10: International classification of diseases 10th revision
MBRN: Medical birth registry of Norway
NOK: Norwegian kroner
NPR: Norwegian patient register
USA: United States of America

## References

[1] Global Research on Developmental Disabilities Collaborators (2018). Developmental disabilities among children younger than 5 years in 195 countries and territories, 1990–2016: a systematic analysis for the global burden of disease study 2016. Lancet Glob Health.

[2] Prevention CfDCa. https://www.cdc.gov/ncbddd/developmentaldisabilities/facts.html. Accessed 27 Oct 2022.

[3] Modabbernia A, Velthorst E, Reichenberg A (2017). Environmental risk factors for autism: an evidence-based review of systematic reviews and meta-analyses. Mol Autism.

[4] Ramaswami G, Geschwind DH (2018). Genetics of autism spectrum disorder. Handb Clin Neurol.

[5] Surén P, Havdahl A, Øyen AS, Schjølberg S, Reichborn-Kjennerud T, Magnus P (2019). Diagnosing autism spectrum disorder among children in Norway. Tidsskr Nor Laegeforen.

[6] Health NIoP. fact sheet ADHD 2015 https://www.fhi.no/fp/psykiskhelse/psykisk-helse-barn-unge/adhd---faktaark/.

[7] van der Ven E, Termorshuizen F, Laan W, Breetvelt EJ, van Os J, Selten JP (2013). An incidence study of diagnosed autism-spectrum disorders among immigrants to the Netherlands. Acta Psychiatr Scand.

[8] Morinaga M, Rai D, Hollander AC, Petros N, Dalman C, Magnusson C (2021). Migration or ethnic minority status and risk of autism spectrum disorders and intellectual disability: systematic review. Eur J Pub Health.

[9] Lehti V, Hinkka-Yli-Salomäki S, Cheslack-Postava K, Gissler M, Brown AS, Sourander A (2013). The risk of childhood autism among second-generation migrants in Finland: a case-control study. BMC Pediatr.

[10] Schmengler H, Cohen D, Tordjman S, Melchior M (2021). Autism spectrum and other neurodevelopmental disorders in children of immigrants: a brief review of current evidence and implications for clinical practice. Front Psychiatry.

[11] Kawa R, Saemundsen E, Lóa Jónsdóttir S, Hellendoorn A, Lemcke S, Canal-Bedia R (2017). European studies on prevalence and risk of autism spectrum disorders according to immigrant status-a review. Eur J Pub Health.

[12] Hjern A, Weitoft GR, Lindblad F (2010). Social adversity predicts ADHD-medication in school children–a national cohort study. Acta paediatr.

[13] Lehti V, Chudal R, Suominen A, Gissler M, Sourander A (2016). Association between immigrant background and ADHD: a nationwide population-based case-control study. J Child Psychol Psychiatry.

[14] Loi S, Pitkänen J, Moustgaard H, Myrskylä M, Martikainen P (2021). Health of immigrant children: the role of immigrant generation, exogamous family setting, and family material and social resources. Demography.

[15] Lehti V, Gyllenberg D, Suominen A, Sourander A (2018). Finnish-born children of immigrants are more likely to be diagnosed with developmental disorders related to speech and language, academic skills and coordination. Acta Paediatr.

[16] Barn og unge i befolkningen [children and adolescences in the population]. 2019. https://www.ssb.no/a/barnogunge/2019/bef/. Accessed 27 Nov 2022.

[17] Moe D, Molstad CS (2022). Standarder for verdensinndelinger og landkoder.

[18] R_Core_Team. R: A Language and Environment for Statistical Computing Vienna, Austria: R Core Team; 2021. https://www.R-project.org/. Accessed 27 Nov 2022.

[19] Borgan Ø (2000). Modeling survival data: Extending the Cox Model Terry M. Therneau and Patricia M. Grambsch.

[20] Kennedy N. Forestmodel: Forest Plots from Regression Models, R Package Version 06. R Core Team. 2020.

[21] Arat A, Östberg V, Burström B, Hjern A (2018). ADHD medication in offspring of immigrants—does the income level of the country of parental origin matter?. BMC Psychiatry.

[22] Bakken KS, Skjeldal OH, Stray-Pedersen B (2015). Higher risk for adverse obstetric outcomes among immigrants of African and Asian descent: a comparison study at a low-risk maternity hospital in Norway. Birth.

[23] Haglund NG, Källén KB (2011). Risk factors for autism and asperger syndrome perinatal factors and migration. Autism Int J Res Pract.

[24] Lauritsen MB, Pedersen CB, Mortensen PB (2005). Effects of familial risk factors and place of birth on the risk of autism: a nationwide register-based study. J Child Psychol Psychiatry.

[25] Augereau N, Lagdas I, Kermarrec S, Gicquel L, Martin V, Xavier J (2020). Premigration social adversity and autism spectrum disorder. BJPsych open.

[26] Magnusson C, Rai D, Goodman A, Lundberg M, Idring S, Svensson A (2012). Migration and autism spectrum disorder: population-based study. Br J Psychiatry.

[27] Paulsrud K AZ, Drangsholt A, Evensen S, Hagen CM, Lund A, Munkhaugen EK, Nøstvik LI, Suren P, Ugelstad H, Vea SO, Weidle B, Østbøll EK, Opsahl K. Tjenester til personer med autismespekterforstyrrelser og til personer med Tourettes syndrom. NOU-Norges offentlige utredninger 2020:1. Helse- og Omsorgsdepartementet 2020.

[28] Franz L, Chambers N, von Isenburg M, de Vries PJ (2017). Autism spectrum disorder in sub-saharan Africa: a comprehensive scoping review. Autism Res.

[29] Crafa D, Warfa N (2015). Maternal migration and autism risk: systematic analysis. Int Rev Psychiatry.

[30] Hambly H, Wren Y, McLeod S, Roulstone S (2013). The influence of bilingualism on speech production: a systematic review. Int J Lang Commun Disord.

[31] Hoff E, Ribot KM (2017). Language growth in english monolingual and Spanish-english bilingual children from 2.5 to 5 years. J pediat.

[32] Trillingsgaard A, Ulsted Sørensen E, Nemec G, Jørgensen M (2005). What distinguishes autism spectrum disorders from other developmental disorders before the age of four years?. Eur Child Adolesc Psychiatry.

