# Supplementary figures and images for: Developmental disorders among Norwegian-born children with immigrant parents
Source: Child Adolesc Psychiatry Ment Health. 2023 Jan 6;17:3. doi: 10.1186/s13034-022-00547-x (PMC9825022; doi:10.1186/s13034-022-00547-x)

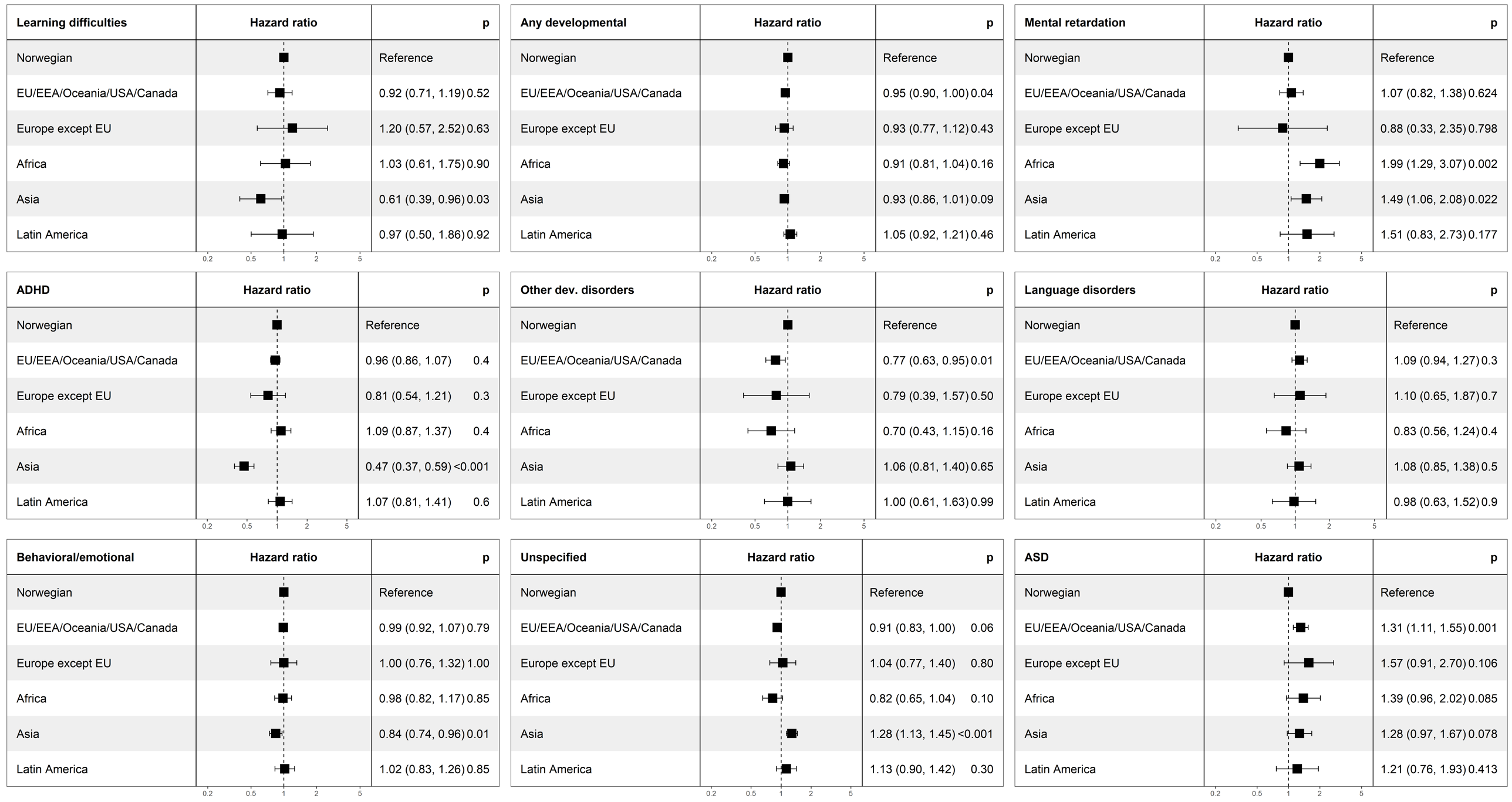

Supplement: Supplementary file 1 — Additional file 1: Figure S1. Hazard ratio for diagnosis of developmental disorder by maternal regional background for children born in Norway between 2006 and 2017 with only one immigrant parent (mother) compared to children with two Norwegian-born parents, adjusted for birth year, sex, highest achieved parental educational level, and household income group (95% confidence interval). [file 13034_2022_547_MOESM1_ESM.tiff]

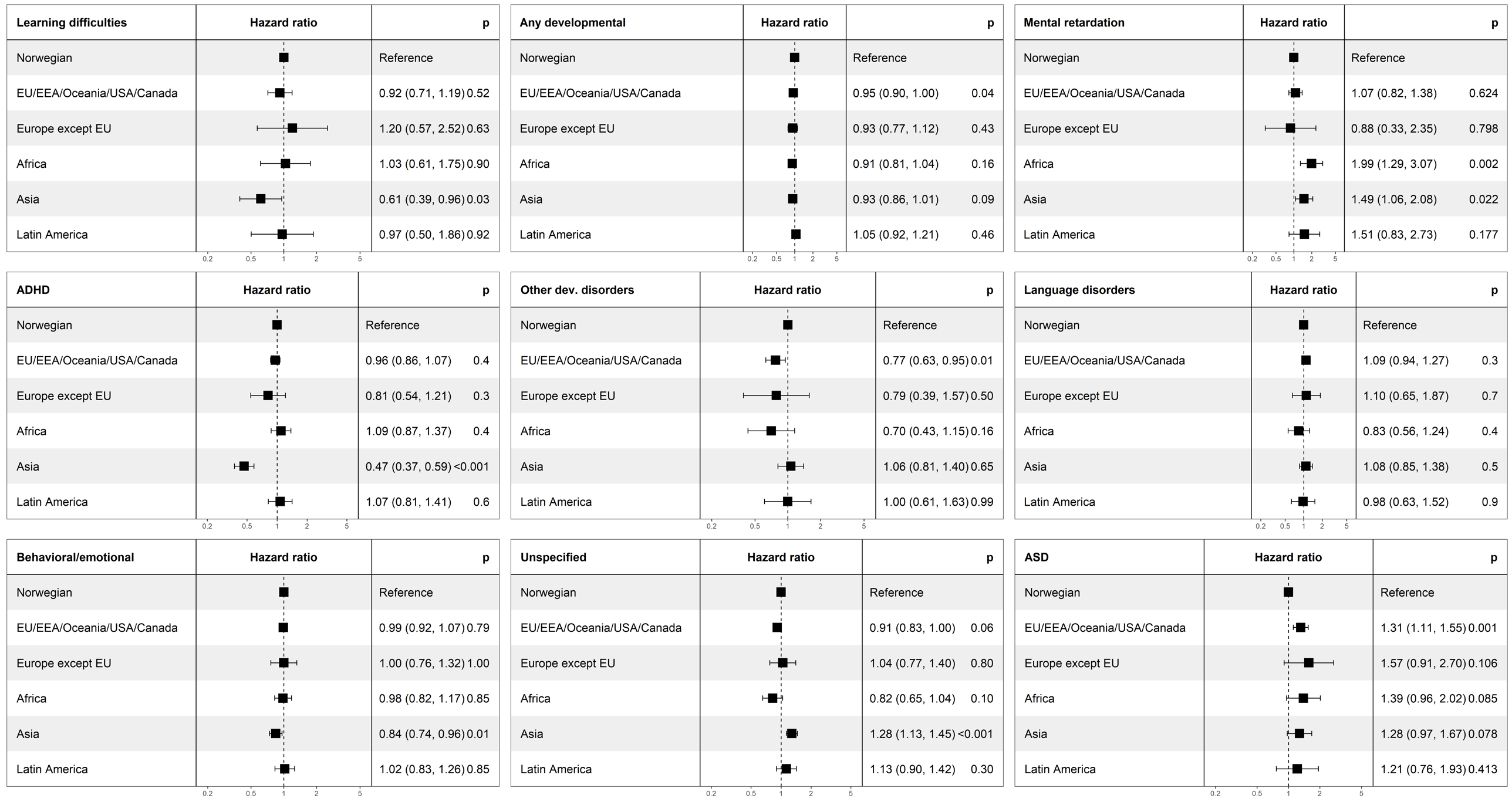

Supplement: Supplementary file 2 — Additional file 2: Figure S2. Hazard ratio for diagnosis of developmental disorder by paternal regional background for children born in Norway between 2006 and 2017 with only one immigrant parent (father) compared to children with two Norwegian-born parents, adjusted for birth year, sex, highest achieved parental educational level, and household income group (95% confidence interval). [file 13034_2022_547_MOESM2_ESM.tiff]

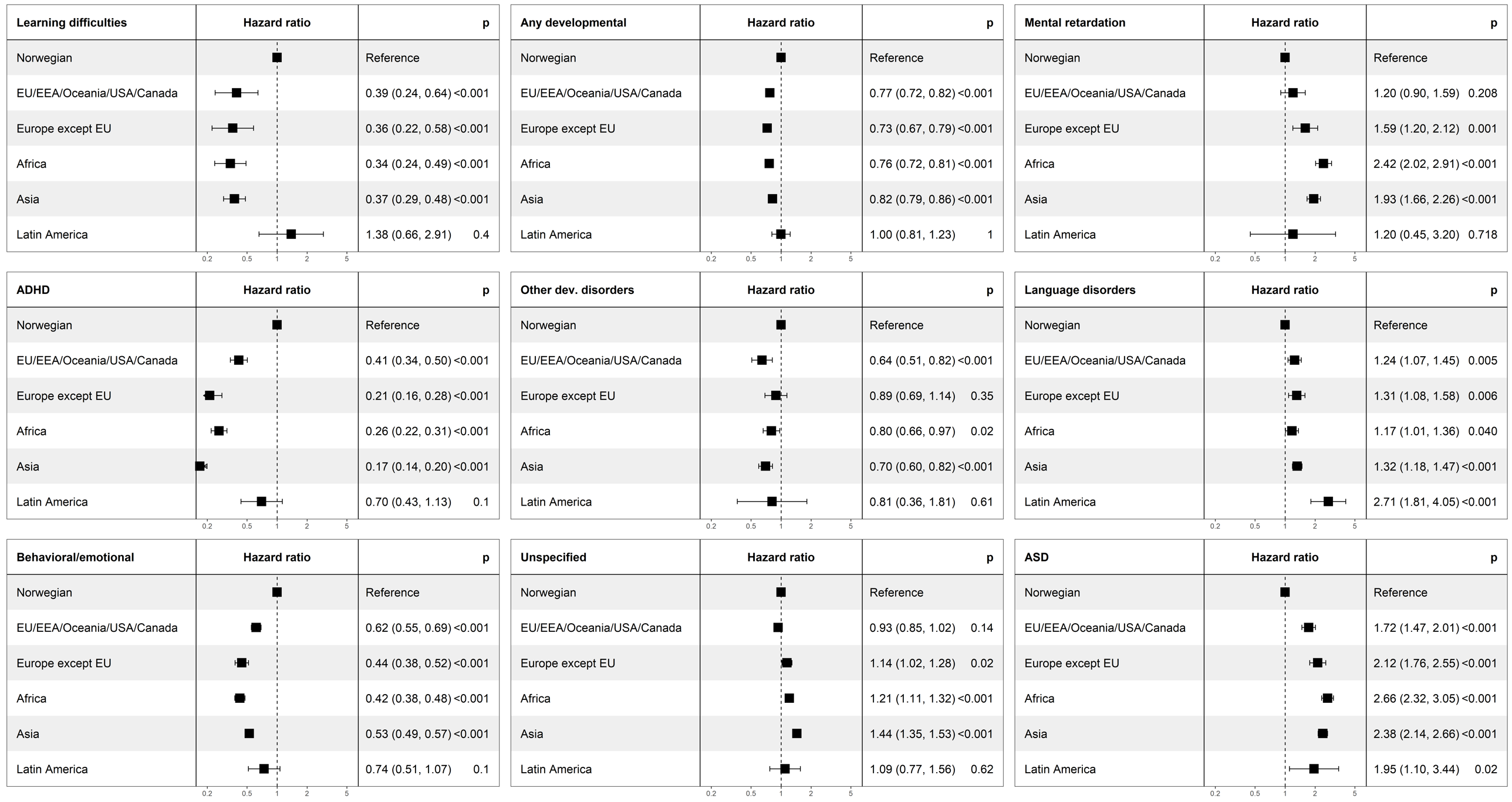

Supplement: Supplementary file 3 — Additional file 3: Figure S3. Hazard ratio for diagnosis of developmental disorder by parental regional background for children born in Norway between 2006 and 2017 with two immigrant parents compared to children with two Norwegian-born parents, adjusted for birth year, sex, highest achieved parental educational level, and household income group (95% confidence interval). [file 13034_2022_547_MOESM3_ESM.tiff]
