# Supplementary material for: Developmental disorders among Norwegian-born children with immigrant parents
Source: Child Adolesc Psychiatry Ment Health. 2023 Jan 6;17:3. doi: 10.1186/s13034-022-00547-x (PMC9825022; doi:10.1186/s13034-022-00547-x)
Supplement: Supplementary file 4 — Additional file 4: Table S1. ICD-10 codes included per diagnostic category. Table S2. Developmental disorder diagnoses given before the age of 6 in secondary/tertiary health care (N (%)) and between 2008 and 2018 among children born in Norway between 2006 and 2017 with two Norwegian-born parents, two immigrant parents and one immigrant parent (mother/father). Table S3. Mean age (year, SD) at diagnosis of developmental disorders given between 2008 and 2018 among children born in Norway between 2006 and 2017. [file 13034_2022_547_MOESM4_ESM.docx]

**Supplementary tables and figures**

Supplementary Table 1 ICD-10 codes included per diagnostic category

| Developmental disorder diagnostic category | ICD-10 CODES |
| --- | --- |
| Mental retardation | F70-F79 |
| Language disorders | F80 |
| Learning difficulties | F81 |
| Other developmental disorders | F82 – F83, F88 – F89 |
| Autism spectrum disorders | F84 |
| ADHD | F90 |
| Behavioral and emotional disorders in childhood, | F91-F98 |
| Unspecified developmental delay | R62 |

Supplementary Table 2 Developmental disorder diagnoses given before the age of 6 in secondary/tertiary health care (N (%)) and between 2008 and 2018 among children born in Norway between 2006 and 2017 with two Norwegian-born parents, two immigrant parents and one immigrant parent (mother/father)

|  | **Two Norwegian-born parents** | **Two immigrant parents** | **EU/EEA/Oceania/USA/Canada** | **Europe except EU** | **Africa** | **Asia** | **Latin America** |
| --- | --- | --- | --- | --- | --- | --- | --- |
| Mental retardation | 500 (0.10) | 224 (0.24) *** | 30 (0.11) | 25 (0.24) *** | 79 (0.40) *** | 89 (0.26) *** |  |
| Language disorders | 2258 (0.46) | 677 (0.72) *** | 144 (0.52) | 77 (0.74) *** | 160 (0.81) *** | 279 (0.82) *** | 17 (1.36) *** |
| Learning difficulties | 39 (0.01) | 10 (0.01) |  |  |  |  |  |
| Other developmental disorders | 1630 (0.33) | 299 (0.32) | 61 (0.22) ** | 39 (0.37) | 77 (0.39) | 118 (0.35) |  |
| Autism spectrum disorders | 1196 (0.25) | 918 (0.98) *** | 154 (0.55) *** | 104 (1.00) *** | 273 (1.38) *** | 375 (1.10) *** | 12 (0.96) *** |
| ADHD | 710 (0.15) | 71 (0.08) *** | 16 (0.06) *** | 6 (0.06) * | 21 (0.11) | 24 (0.07) *** |  |
| Behavioral and emotional disorders in childhood, | 4404 (0.90) | 749 (0.80) ** | 141 (0.51) *** | 74 (0.71) * | 145 (0.73) * | 375 (1.10) *** | 14 (1.12) |
| Unspecified developmental delay | 9555 (1.96) | 2572 (2.75) *** | 523 (1.88) | 275 (2.63) *** | 602 (3.05) *** | 1145 (3.35) *** | 27 (2.16) |
| Any developmental disorder | 16545 (3.39) | 4183 (4.48) *** | 825 (2.97) *** | 461 (4.42) *** | 996 (5.04) *** | 1841 (5.38) *** | 60 (4.79) *** |
|  |  | **Norwegian-born father and immigrant mother** | **EU/EEA/Oceania/USA/Canada** | **Europe except EU** | **Africa** | **Asia** | **Latin America** |
| Mental retardation |  | 61 (0.12) | 21 (0.09) |  |  | 26 (0.16) * | 7 (0.15) |
| Language disorders |  | 274 (0.55) ** | 93 (0.42) | 30 (0.67) | 14 (0.61) | 95 (0.60) * | 42 (0.93) *** |
| Learning difficulties |  |  |  |  |  |  |  |
| Other developmental disorders |  | 157 (0.32) | 69 (0.31) | 13 (0.29) | 7 (0.30) | 52 (0.33) | 16 (0.35) |
| Autism spectrum disorders |  | 259 (0.52) *** | 78 (0.35) ** | 32 (0.72) *** | 14 (0.61) ** | 105 (0.66) *** | 30 (0.66) *** |
| ADHD |  | 51 (0.10) * | 32 (0.14) | 5 (0.11) |  | 5 (0.03) *** | 6 (0.13) |
| Behavioral and emotional disorders in childhood, |  | 464 (0.94) | 194 (0.87) | 43 (0.96) | 31 (1.34) * | 139 (0.87) | 57 (1.26) * |
| Unspecified developmental delay |  | 1075 (2.17) ** | 446 (2.00) | 103 (2.31) | 40 (1.73) | 387 (2.43) *** | 99 (2.18) |
| Any developmental disorder |  | 1873 (3.78) *** | 758 (3.40) *** | 186 (4.17) *** | 89 (3.85) *** | 639 (4.01) *** | 201 (4.43) *** |
|  |  | **Norwegian-born mother and immigrant father** | **EU/EEA/Oceania/USA/Canada** | **Europe except EU** | **Africa** | **Asia** | **Latin America** |
| Mental retardation |  | 55 (0.14) * | 29 (0.12) |  | 6 (0.17) | 15 (0.20) * |  |
| Language disorders |  | 210 (0.53) | 120 (0.50) | 10 (0.59) | 16 (0.46) | 52 (0.69) ** | 12 (0.44) |
| Learning difficulties |  |  |  |  |  |  |  |
| Other developmental disorders |  | 120 (0.31) | 58 (0.24) * | 5 (0.29) | 12 (0.34) | 36 (0.48) * | 9 (0.33) |
| Autism spectrum disorders |  | 157 (0.40) *** | 85 (0.36) ** | 8 (0.47) | 15 (0.43) * | 41 (0.55) *** | 8 (0.29) |
| ADHD |  | 47 (0.12) | 25 (0.10) |  | 6 (0.17) | 12 (0.16) |  |
| Behavioral and emotional disorders in childhood, |  | 363 (0.92) | 201 (0.84) | 12 (0.71) | 41 (1.17) | 84 (1.12) | 25 (0.91) |
| Unspecified developmental delay |  | 783 (1.99) | 409 (1.71) ** | 39 (2.30) | 55 (1.57) | 216 (2.89) *** | 64 (2.34) |
| Any developmental disorder |  | 1351 (3.44) *** | 721 (3.02) *** | 60 (3.54) | 114 (3.26) | 354 (4.73) | 102 (3.73) |

N<5 not shown. Star indicting difference to having two Norwegian-born parents: ***p<0.001 **p<0.01 *p<0.05

Supplementary Table 3 Mean age (year, SD) at diagnosis of developmental disorders given between 2008 and 2018 among children born in Norway between 2006 and 2017

|  | **Two Norwegian-born parents** | **Two immigrant parents** | **EU/EEA/**  **Oceania/USA/Canada** | **Europe except EU** | **Asia** | **Africa** | **Latin America** |
| --- | --- | --- | --- | --- | --- | --- | --- |
| Mental retardation | 6.3 (2.6) | 5.8 (2.4) | 5.1 (2.4) | 5.7 (2.2) | 5.7 (2.3) | 6.1 (2.5) | 7 (2.9) |
| Language disorders | 5.2 (2.4) | 4.7 (2.1) | 4.6 (1.8) | 5.1 (2.1) | 4.5 (1.9) | 4.7 (2.2) | 4.9 (1.7) |
| Learning difficulties | 9.2 (1.7) | 8.3 (2) | 7.8 (1.7) | 8.1 (1.6) | 8.4 (2.4) | 8.4 (2) | 8.4 (2.6) |
| Other developmental disorders | 4.7 (3.2) | 4.4 (3.1) | 3.4 (2.7) | 5.1 (3.3) | 4.5 (2.9) | 4.5 (3.1) | 4.7 (3.3) |
| Autism spectrum disorders | 6.2 (2.8) | 4.1 (1.8) | 4.4 (2) | 4.2 (1.8) | 3.9 (1.6) | 4.1 (1.9) | 3.6 (1.3) |
| ADHD | 8.1 (1.9) | 7.5 (2) | 7.5 (2.1) | 7.9 (2) | 7.4 (1.9) | 7.5 (2.1) | 7.6 (1.9) |
| Behavioral and emotional disorders | 6.8 (2.5) | 5.4 (2.9) | 5.8 (2.7) | 5.7 (2.7) | 5.6 (2.8) | 5.1 (3) | 5.8 (2.6) |
| Unspecified developmental delay | 2.9 (2.5) | 2.8 (2.3) | 2.4 (2) | 2.8 (2.2) | 2.9 (2.2) | 2.9 (2.5) | 3.3 (2.1) |
| Any developmental disorder | 5.5 (3.2) | 3.9 (2.7) | 3.9 (2.8) | 4.1 (2.7) | 3.9 (2.6) | 3.8 (2.8) | 4.7 (2.7) |
|  |  | **Norwegian-born father and immigrant mother** | **EU/EEA/**  **Oceania/USA/Canada** | **Europe except EU** | **Asia** | **Africa** | **Latin America** |
| Mental retardation |  | 6 (2.4) | 6 (2.6) | 6.6 (2.3) | 5.2 (3.7) | 5.8 (2.3) | 6.4 (2.4) |
| Language disorders |  | 5.1 (2.3) | 5.4 (2.3) | 5.4 (2.5) | 4.4 (1.9) | 4.8 (2.3) | 4.9 (2.1) |
| Learning difficulties |  | 9 (1.9) | 9 (2.1) | 8.3 (2.1) | 7.8 (1.3) | 9.2 (1.7) | 8.8 (1.2) |
| Other developmental disorders |  | 4.5 (3.2) | 4.9 (3.2) | 5.1 (3.1) | 4.8 (3.6) | 4 (3) | 3.4 (3) |
| Autism spectrum disorders |  | 5.2 (2.5) | 5.7 (2.8) | 5.5 (2.5) | 5 (2.3) | 4.7 (2.2) | 5 (2.3) |
| ADHD |  | 7.9 (1.9) | 7.8 (2) | 7.8 (1.8) | 7.7 (2.3) | 8.3 (1.8) | 7.9 (1.7) |
| Behavioral and emotional disorders |  | 6.4 (2.7) | 6.6 (2.7) | 6.7 (2.5) | 6.3 (2.7) | 6.1 (2.7) | 6.4 (2.5) |
| Unspecified developmental delay |  | 2.8 (2.4) | 2.8 (2.6) | 2.9 (2.3) | 2.3 (1.8) | 2.9 (2.3) | 2.7 (2.4) |
| Any developmental disorder |  | 5 (3.1) | 5.2 (3.2) | 5.2 (2.9) | 4.9 (3.1) | 4.5 (3) | 5.1 (3) |
|  |  | **Norwegian-born mother and immigrant father** | **EU/EEA/**  **Oceania/USA/Canada** | **Europe except EU** | **Asia** | **Africa** | **Latin America** |
| Mental retardation |  | 5.8 (2.4) | 5.3 (2.4) | 7 (2.7) | 6.8 (2.4) | 5.9 (2.1) | 5.9 (2.2) |
| Language disorders |  | 4.7 (2.2) | 4.8 (2.1) | 4.6 (1.9) | 4.5 (2.6) | 4.4 (2) | 5.4 (2.8) |
| Learning difficulties |  | 9 (1.6) | 9 (1.6) | 9.3 (1.1) | 8.9 (2) | 8.6 (1.3) | 9.7 (1.1) |
| Other developmental disorders |  | 4.3 (3) | 4.4 (3.1) | 4.1 (2.7) | 4 (3.1) | 4 (2.7) | 4.8 (3.4) |
| Autism spectrum disorders |  | 5.4 (2.6) | 5.5 (2.5) | 5.5 (2.6) | 5.9 (3.1) | 4.5 (2.4) | 5.7 (2.5) |
| ADHD |  | 8 (1.9) | 8 (1.9) | 8 (1.9) | 8 (1.8) | 7.9 (2.3) | 8.3 (1.9) |
| Behavioral and emotional disorders |  | 6.4 (2.6) | 6.5 (2.5) | 6.6 (2.2) | 6.6 (2.6) | 6.1 (2.9) | 6.3 (2.5) |
| Unspecified developmental delay |  | 2.9 (2.5) | 3 (2.5) | 2.6 (2.2) | 3.4 (2.9) | 2.8 (2.4) | 2.7 (2.7) |
| Any developmental disorder |  | 5.2 (3.1) | 5.4 (3.1) | 4.9 (2.9) | 5.7 (3.1) | 4.6 (3.1) | 5.1 (3.1) |
